# A Compost Treatment Acts as a Suppressive Agent in *Phytophthora capsici – Cucurbita pepo* Pathosystem by Modifying the Rhizosphere Microbiota

**DOI:** 10.3389/fpls.2020.00885

**Published:** 2020-06-24

**Authors:** Alessio Bellini, Ilario Ferrocino, Maria Alexandra Cucu, Massimo Pugliese, Angelo Garibaldi, Maria Lodovica Gullino

**Affiliations:** ^1^AGROINNOVA – Centre of Competence for the Innovation in the Agro-Environmental Sector, University of Turin, Turin, Italy; ^2^Agricultural, Forestry and Food Sciences Department (DISAFA), University of Turin, Turin, Italy; ^3^AgriNewTech s.r.l., Turin, Italy

**Keywords:** *Phytophthora capsici*, *Cucurbita pepo*, compost, *Trichoderma*, soil metataxonomy, mycobiota

## Abstract

*Phytophthora capsici* Leonian (PHC) is a filamentous pathogen oomycete that causes root, fruit, foliar and crown rot over a wide host range, including the economically and nutritionally important summer squash (*Cucurbita pepo* var. *cylindrica* L.) crop. PHC chemical control strategies are difficult to adopt, due to the limited number of registered chemicals that are permitted and the scalar harvest system. For these reasons, other strategies, such as the use of waste-based composts that can act as suppressive agents against several soilborne pathogens, have been studied intensively. It is well known that compost’s microbiota plays an important role to confer its suppressive ability. In this study, four different composts were analyzed with both 16S rRNA gene and 18S rRNA gene real-time PCR amplification and with 26S gene amplicon-based sequencing; the total abundance of the bacterial and fungal communities was found to be higher compared to literature, thus confirming that the four composts were a good inoculum source for agricultural applications. The core mycobiota was mainly composed of 31 genera; nevertheless, it was possible to observe a clear predominance of the same few taxa in all the composts. The four composts were then tested, at different concentrations (1–10–20% v/v), to establish their ability to confer suppressiveness to the *Phytophthora capsici* (PHC) – *Cucurbita pepo* pathosystem in controlled greenhouse pot trials. A total of 12 compost mixtures were considered, and of these, one (*Trichoderma*-enriched compost at 10% v/v) was able to statistically reduce the disease incidence caused by PHC (by 50% compared to the untreated control). Hence, the microbiota composition of the most effective compost treatment was investigated and compared with untreated and chemical (metalaxyl) controls. Mycobiota sequencing showed genera differences between the three treatments, with relative abundances of several fungal genera that were significantly different among the samples. Moreover, PCA analyses clustered the compost treatment differently from the chemical and the untreated controls. These findings suggest that suppressive activity of a compost is strictly influenced by its microbiota and the applied dosage, but the ability to induce a shaping in the rhizosphere microbial composition is also required.

## Introduction

*Phytophthora capsici* Leonian (PHC) is a filamentous pathogen oomycete that causes root, fruit, foliar, and crown rot over a wide host range, including some economically and nutritionally important horticultural crops (cucurbits, tomatoes, pepper, eggplant) (Lamour et al., 2012). In Italy, and throughout the world, summer squash (*Cucurbita pepo* var. *cylindrica* L.) cultivation is strongly affected by the natural and human-derived presence of PHC inoculum (Erwin and Ribeiro, 1996). Chemical control strategies are difficult to adopt, due to the limited number of registered chemicals that are permitted and the scalar harvest system (Gilardi et al., 2015). However, many efforts have been made to find a source of genetic resistance in squash accessions to PHC strains (Michael et al., 2019; Siddique et al., 2019), but this approach is still at its beginning. For these reasons, alternative PHC control agents are being studied and adopted in agriculture. Moreover, one of the most promising and most studied technique to prevent the infection of soil-borne pathogens is the application of organic amendments (Meghvansi and Varma, 2015; Gilardi et al., 2016; De Corato et al., 2018a, b).

In the last few decades, interest in using organic amendments has grown throughout the world, due to the possibility of reintroducing recycled biowastes and organic matter into the primary production industry, which is connected to the concept of circular economy. Among the various organic amendments, compost has been studied the most and has been used because of its suppressiveness activity (Pugliese et al., 2015; Bonanomi et al., 2018). The phenomenon of suppressiveness is the ability to limit or avoid the spread of a disease where both susceptible crop variety and pathogen are present in the field; this is connected with the available amount or the addition of organic matter and the ability of the seeds and roots to establish a connection with diverse microorganisms present in the soil that uptake exudates and, consequently, limit the outbreak of pathogens and parasites (Topalović et al., 2020).

Moreover, plant root exudates have been shown to have the ability to select a specific microbiota and influence the colonization of root areas by microorganisms; this means that rhizosphere microbiota is closely connected not only to the soil but also to the plant genotype since different cultivars were shown to be able to select different microbiota (Weller et al., 2002; Haas and Défago, 2005; Bulgarelli et al., 2012; Doornbos et al., 2012; Badri et al., 2013; Mazzola et al., 2015).

Nevertheless, since each compost can be different, suppressive action is not always guaranteed, and, sometimes, a compost can even play a conductive role. Bonanomi et al. (2007) redacted a list of 2,423 articles in this field and showed that only 54% of the studied composts were able to induce suppressiveness. It is also known that a mature compost can play both a suppressive and a conductive role as a function of the pathosystem (Bonanomi et al., 2010). Compost microbiota plays a major role in the suppressive activity of composts, and in an attempt to prove this, many studies have compared sterilized composts with their non-sterilized counterparts and have found that the inactivation of microbiota is connected to the loss of suppressive activity (Reuveni et al., 2002; Tilston et al., 2002; Papasotiriou et al., 2013; De Corato et al., 2019). The microorganisms present in a compost are also selected by root exudates, according to the need and the genotype of the plant, resulting in the shaping of the rhizosphere composition and accomplishing the suppressive activity against several biotic stresses such as fungi and nematodes (Antoniou et al., 2017; Mwaheb et al., 2017; Cucu et al., 2019; Zhang et al., 2020). Thus, in order to prevent soil-borne diseases when pathogens are present in field, it is very important to know which microorganisms confer compost suppressiveness according to the type of pathosystem. Many studies have investigated the microbial communities present in several composts, but most of them were based on *in vitro* isolation, which has the drawback of excluding non-cultivable microorganisms and therefore of not giving a full picture of the entire complexity. Moreover, recent studies have pointed out the importance of using molecular cultural independent methods (Šišić et al., 2018; Cao et al., 2019; Carrasco et al., 2019; Fernández-Bayo et al., 2019; Zhao et al., 2019; Zhou G. et al., 2019).

The objectives of this work were to investigate the microbiota populations of four different composts using targeting (real-time PCR) and non-targeting (amplicon-based Illumina sequencing) molecular approaches; to test their ability to confer suppressiveness in a squash – PHC pathosystem in controlled greenhouse pot trials; and to investigate, on the basis of the greenhouse trial results, the microbiota composition of the best compost treatment, in comparison with an untreated and a chemical treatment, by means of the same molecular tools used for the compost characterization.

## Materials and Methods

### Composts Used in This Study

Four different commercial composts were used in this study: (i) a green waste compost produced in a dynamic composting system for 6 months and sifted with a 10 mm sieve (ANT’s Compost V – CV; AgriNewTech s.r.l., Italy), (ii) the same green compost enriched with experimental BCA “*Trichoderma* sp. TW2” (ANT’s compost M – CM; AgriNewTech s.r.l., Italy), (iii) a municipal biowaste compost produced using green and urban organic fraction biowastes in a dynamic composting system for 4 months (ANT’s Compost B – CB; AgriNewTech s.r.l., Italy), and (iv) a green compost produced in a dynamic composting system for 6 months and sifted with a 20 mm sieve (ANT’s compost V2 – CV2; AgriNewTech s.r.l., Italy). At the end of the maturation process, CV, CM, CV2, and CB were analyzed by an external laboratory to establish their chemical compositions (Table 1).

**TABLE 1:** Chemical composition of the tested composts.

	**CV/CM**	**CV2**	**CB**
pH	7.92	8.08	8.08
Humidity (%)	42.00	43.10	40.90
Organic C (g/kg dry matter)	210.00	258.00	220.00
Organic N (g/kg dry matter)	15.70	23.60	21.30
C/N ratio	13.00	9.44	9.16
Total N (g/kg dry matter)	16.30	15.50	24.00
Organic N/total N ratio	96.00	88.45	88.75
Hg (mg/kg dry matter)	0.16	<0.01	<1.50
Ni (mg/kg dry matter)	93.10	11.6	84.00
Pb (mg/kg dry matter)	47.90	32.1	39.00
Zn (mg/kg dry matter)	143.80	140.10	206.00
Cu (mg/kg dry matter)	52.50	56.60	148.00

### Greenhouse Trials

Summer squash (*Cucurbita pepo* var. *cylindrica* L. cv Genovese) seeds were sown in seed cells in a peat substrate (Tecno 2, 70% white peat and 30% clay, pH 5.5–6, N 110–190 mg/L, P_2_O_5_ 140–230 mg/L, K_2_O 170–280 mg/L, Turco Silvestro terricci, Bastia d’Albenga, SV, Italy) and kept in a nursery for 2 weeks at 26 ± 1°C. In the meantime, substrates were prepared for potted plants, by adding different percentage of each compost (1–10–20% v/v) to the same peat used for sowing. After 1 week, each substrate mixture was infested with 2 g/l of fresh biomass of one strain of PHC (AGROINNOVA collection), grown for 2 weeks in grain-hemp (60:40) flasks, according to the method described in Gilardi et al. (2015). A chemical treatment, in which a suspension of metalaxyl (Ridomil gold, 480 g/l, Syngenta Crop Protection) and water was used in order to reach a final concentration of 50 μl/l of substrate, was carried out at the same time as the inoculation. One week after the infestation, the seedlings were transplanted into 21 pots, with 3 plants per pot, and placed in a greenhouse kept at 24 ± 1°C. Each treatment was replicated in three different pots per trial, with a randomized experimental design. The experiment was carried out twice independently.

### Disease Assessment

Disease incidence (DI) was evaluated by counting the number of diseased plants in each pot twice during the trials, according to the formula: $\frac{\mathrm{number}\,\mathrm{of}\,\mathrm{diseased}\,\mathrm{plants}}{\mathrm{number}\,\mathrm{of}\,\mathrm{total}\,\mathrm{plants}}\times 100$; an intermediate disease assessment was performed 1 week after transplantation; the final evaluation was performed 1 week later. The fresh biomass of the plants was also weighed. The area under the disease progress curve (AUDPC) was calculated according to Pandey et al. (1989).

### Sampling and DNA Extraction

Each compost was collected individually from different and random parts of big bags (total volume 50 ml). Two separate DNA extractions were carried out for each compost using 100 mg of fresh compost. The rhizosphere was collected at the end of the pot trials. Three biological replicates were collected from three different pots per treatment and per trial, the plant roots were shaken to remove any excess peat, and the particles that were still adhered to the root system were collected in 50 ml vials. Total microbial DNA extraction was carried out with EZNA soil DNA kit using 100 mg of soil (Omega Bio-Tek, Norcross, GA, United States), following manufacturer’s instructions. DNA quantity was assessed using a NanoDrop 2000 spectrophotometer (Thermo Fisher Scientific, Waltham, MA, United States), while DNA integrity was verified by running 5 μl of each sample in a 1% agarose electrophoretic gel.

### Real-Time PCR Assays

Real-time PCR assays were performed using a StepOne-Plus^TM^ Real-Time System (Applied Biosystems, Foster City, CA, United States). The abundance of the total fungal (18S rRNA gene) and bacterial (16S rRNA gene) communities in the compost samples was determined. The abundance in the rhizosphere samples was instead assessed, for the total fungi, total bacteria, and *Phytophthora capsici*, with the primers by Lan et al. (2013) and under the conditions described by Cucu et al. (2020). Real-time PCR was performed for each extraction in triplicate, and the average values were then transformed into Log of gene copies per gram of dry compost; these data were mediated between two extractions of each sample. Table 2 summarizes the primers and real-time PCR conditions.

**TABLE 2:** Description of the primer sets and amplification conditions of the quantitative real-time PCR assays.

**Gene**	**Primers**	**Real-time PCR conditions**
18S rRNA gene, total fungal abundance	FR1 (Vainio and Hantula, 2000) 390FF (Vainio and Hantula, 2000)	45 cycles 95°C 30”, 50°C 30”, 70°C 60”
16S rRNA gene, total bacterial abundance	Eub338 (Muyzer et al., 1993) Eub51 (Muyzer et al., 1993)	40 cycles 95°C 30”, 55°C 35”, 72°C 45”
*Phytophthora capsici*, pathogen abundance	Pc1F (Lan et al., 2013) Pc1R (Lan et al., 2013)	40 cycles 95°C 30”, 60°C 35”, 72°C 45”

### Amplicon-Based Sequencing

The mycobiota were evaluated by amplifying the D1 domain of the 26S gene using the primers and condition described by Mota-Gutierrez et al. (2019). A library preparation was performed according to the Illumina metagenomic procedure. Sequencing was performed using a MiSeq instrument (Illumina) with V3 chemistry and 250-bp generated paired-end reads, following the manufacturer’s instructions. After sequencing, reads were assembled, quality filtered and processed using QIIME 1.9.0 software (Caporaso), and the pipeline described by Mota-Gutierrez et al. (2019). Centroids sequences of each cluster were manually checked by Blast tool to confirm the taxonomic assignment. QIIME was used to rarefy the OTU table at the lowest number of sequences per sample and to build the OTU table. The OTU table displays the highest taxonomy resolution that was reached; when the taxonomy assignment was not able to reach the genus level, family name was displayed. Relative abundance of OTUs was used to build a principal component analysis (PCA) as a function of the treatment. Anosim statistical test was used, through the *vegan* function of R, to identify any significant differences as a function of the treatments. α-diversity was assessed by Chao1 index, estimating the number of different taxa, and by Shannon diversity index, evaluating the taxa richness calculated using the diversity function of the vegan package in R environment. The Wilcoxon matched pairs test was used to establish the difference in OTUs abundance as a function of the treatment. *P*-values were adjusted for multiple testing using the Benjamini–Hochberg procedure, which assesses the false discovery rate (FDR).

### Statistical Analyses

Statistical analyses were performed, with SPSS software (IBM SPSS Statistics, Westland, MI, United States), for the disease incidence, fresh biomass, AUDPC, and real-time PCR data. ANOVA and Tukey’s *post hoc* tests were performed to establish the statistical values of the differences (*P* < 0.05). The DI, fresh biomass, and AUDPC data were unified for the two separate trials.

### Availability of the Sequence Data

The sequencing data were deposited at the Sequence Read Archive of the National Centre for Biotechnology Information under BioProject number PRJNA580394.

## Results

### Abundance of the Total Microbial Community in Four Composts

**FIGURE 1:**
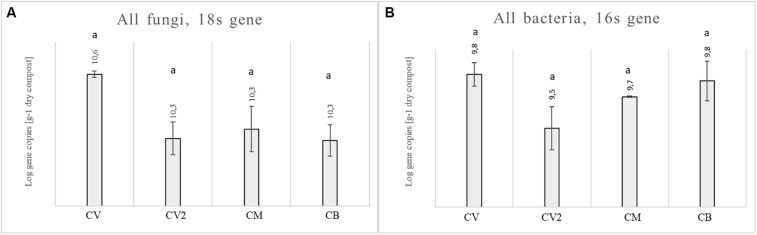
Abundance of the fungal 18S rRNA gene **(A)** and bacterial 16S rRNA gene **(B)** for four composts: CV, CM, CB, and CV2. Different letters indicate statistical differences between the four composts, as obtained with the ANOVA test and Tukey’s *post hoc* test.

Analyses were carried out, with real-time PCR, to describe the microbiological assessment in four composts, in terms of gene abundance expressed as Log of copy^–1^ numbers per gram of dry matter (Figures 1A,B). The fungal 18S rRNA gene copies per 1 g of dry compost were between 10.29 and 10.56 for the four composts, while the bacterial 16S rRNA gene copies were between 9.48 and 9.84. No statistical differences were observed for the fungal or bacterial communities, since the four composts showed a similar abundance.

### Mycobiota Composition of the Four Composts Used in This Study

**FIGURE 2:**
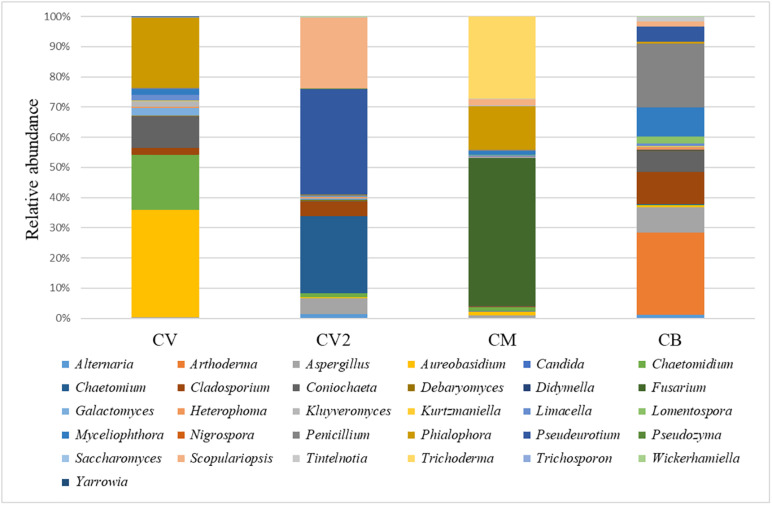
Relative abundance of the fungal community in the four analyzed composts: CV green compost, CV2 green compost, CM green compost with the addition of *Trichoderma* sp. TW2 and CB mixed compost. The OTUs were selected by discarding the ones that were not present in the four composts under a threshold of 0.5%.

A total of 244,298 raw reads (2 × 250 bp) were obtained after sequencing. After quality filtering, a total of 242,702 clean reads were used, with an average value of 60,675 reads/sample and an average sequence length of 386 bp. 26S rRNA gene sequencing showed differences between the four composts used in this study at a genus level. Thirty-one genera were detected (Figure 2 and Supplementary Table S1), and it was possible to observe a clear predominance of a few taxa in all the composts. CV was mostly populated by *Phialophora* (5.5%), *Coniochaeta* (4.2%), and *Aureobasidium* (8.5%); CV2 showed *Penicillium* (21.1%), *Myceliophthora (9.8%)*, *Coniochaeta* (2.5%), *Cladosporium* (10.7%), *Aspergillus* (8.5%), *Arthoderma* (13.3%), and *Pseudoeurotium* (5%); CB showed *Scopulariopsis* (2.6%), *Pseudeurotium* (4%), and *Chaetomium* (2.9%). CM was mostly populated by three main genera: *Trichoderma* (6.4%), *Phialophora* (3.4%), and *Fusarium* (11.5%).

### Disease Suppression by the Compost Mixtures

The negative control – non-inoculated (NC) and chemical control (CC) showed no disease symptoms at the end of both trials. The inoculated untreated control (UC) showed up to 90% of disease incidence at the end of the trials. All the treatments showed a numerical reduction in DI, compared to the UC, and all the CM mixtures had the lowest disease index, but only samples from CM – 10% showed a statistical reduction of DI, compared to the UC (*P* < 0.05), that ranged from 90 to 45%. As far as the fresh biomass is concerned, the untreated control showed the lowest value of all (5.8 ± 1.9 g), while the highest was for the non-inoculated treatment (34.0 ± 2.4 g). Of all the compost mixtures, CM – 10% was the only one that was significantly different from the UC, with an average fresh biomass of 26.3 g (*P* < 0.05).

The area under the disease progress curve (AUDPC) values highlighted a similar situation as the DI and fresh biomass: the inoculated untreated control (UC) had the highest AUDPC value (351) of all the treatments, but only CM – 10% was significantly different from UC, with a value of 137. All the data are shown in Table 3, where the results of Trial 1 and Trial 2 were averaged.

**TABLE 3:** Efficacy of the compost mixtures to suppress *Phytophthora capsici* disease on summer squash plants expressed as disease incidence (%), fresh biomass (g), and AUDPC values.

**Treatments**	**Disease incidence**	**Standard error**	**Tukey**	**AUDPC**	**Standard error**	**Tukey**	**Fresh biomass**	**Standard error**	**Tukey**
Non-inoculated control	0.0	0.0	c	0.0	0.0	a	34.0	2.4	d
Untreated control	90.0	4.7	a	351.3	28.8	c	5.8	1.9	a
Chemical control	0.0	0.0	c	0.0	0.0	a	32.8	3.1	cd
CV – 1%	70.0	9.4	ab	268.8	47.3	bc	9.8	3.0	ab
CV – 10%	75.0	12.5	ab	301.3	49.1	bc	17.6	5.0	abc
CV – 20%	57.5	11.6	ab	212.9	48.6	abc	21.2	2.9	abc
CV2 – 1%	72.5	13.3	ab	287.5	58.2	bc	10.6	4.2	ab
CV2 – 10%	75.0	10.3	ab	286.3	44.9	bc	17.5	3.5	abc
CV2 – 20%	70.0	6.3	ab	257.5	30.6	bc	18.7	4.3	abc
CM – 1%	55.0	9.4	ab	245.4	44.0	bc	12.4	3.1	ab
CM – 10%	45.0	9.1	b	137.1	36.7	ab	26.3	3.3	bcd
CM – 20%	55.0	12.9	ab	181.3	49.1	abc	21.7	2.9	abc
CB – 1%	67.5	10.6	ab	251.7	41.2	bc	16.6	2.3	abc
CB – 10%	67.5	6.6	ab	226.7	30.7	bc	21.5	2.7	abc
CB – 20%	72.5	10.3	ab	286.3	42.8	bc	18.1	5.9	abc

*The letters refer to the Tukey’s *post hoc* test, which was performed after one-way ANOVA (*P* < 0.05).*

### Abundance of Fungi, Bacteria, and PHC in the Rhizosphere of UC, CC, and CM – 10%

**FIGURE 3:**
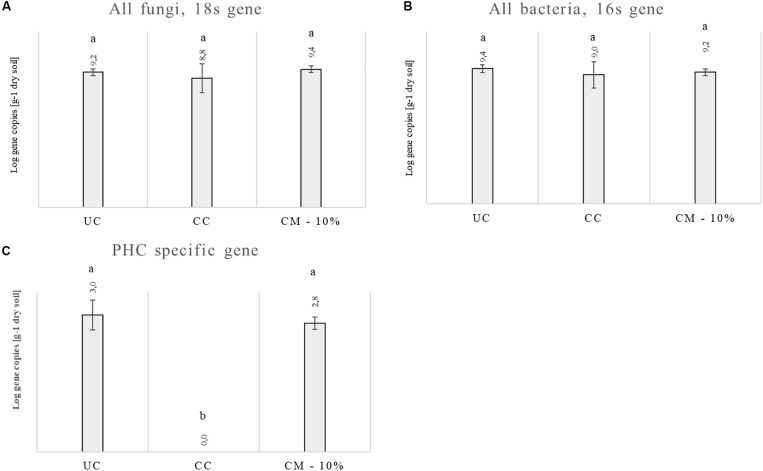
Abundance of the fungal 18S rRNA gene **(A)**, bacterial 16s rRNA gene **(B),** and PHC specific gene **(C)** in the rhizosphere samples at the end of the trials: untreated control (UC), chemical control (CC), and CM – 10% treatment. Three biological replicates were collected from three different pots per treatment and for each trial. PHC means *Phytophthora capsici*. Data from the two trials were analyzed separately and then averaged. Different letters indicate statistical differences between the treatments obtained with the ANOVA test and the Tukey’s *post hoc* test.

Since the only effective treatment was CM – 10%, molecular analyses were carried out using rhizosphere soils collected at the end of the trials for the untreated control, chemical control, and CM – 10% treatments. Data from two trials are shown in Figures 3A–C. No differences were evident for the abundance of fungi and bacteria between the three treatments. The specific PHC gene was found in both the untreated control and in the CM – 10% treatment at levels of 3 and 2.82, respectively [Log of copy^–1^ numbers per gram of dry matter], while it was not found in the chemical treatment.

### Mycobiota Composition of the Rhizosphere Soils

The total number of paired sequences obtained from samples reached 884,148 raw reads. A total of 784,797 reads were obtained after quality filtering, with an average value of 35,672 ± 17,772 reads/sample and a mean sequence length of 393 bp. The α-diversity index showed a satisfactory coverage for all the samples (>96%), but did not show a different level of complexity on the basis of the treatment. No significant difference in mycobiota composition or in α-diversity index was observed (Supplementary Figure S1), and the data were therefore averaged.

**FIGURE 4:**
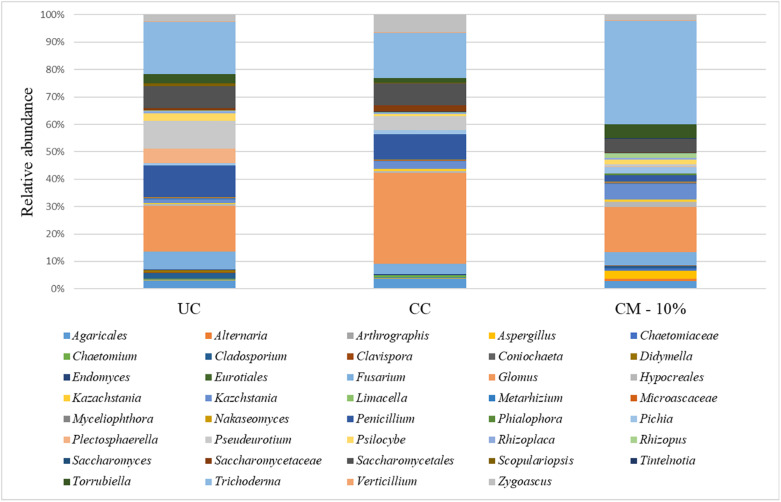
Relative abundance of the mycobiota in the rhizosphere samples at the end of the trials: untreated control (UC), chemical control (CC), and CM – 10% treatment. Three biological replicates were collected from three different pots per treatment and for each trial. Only OTUs which showed an incidence above 0.2% in at least two samples are shown. The data from replicates were averaged.

**FIGURE 5:**
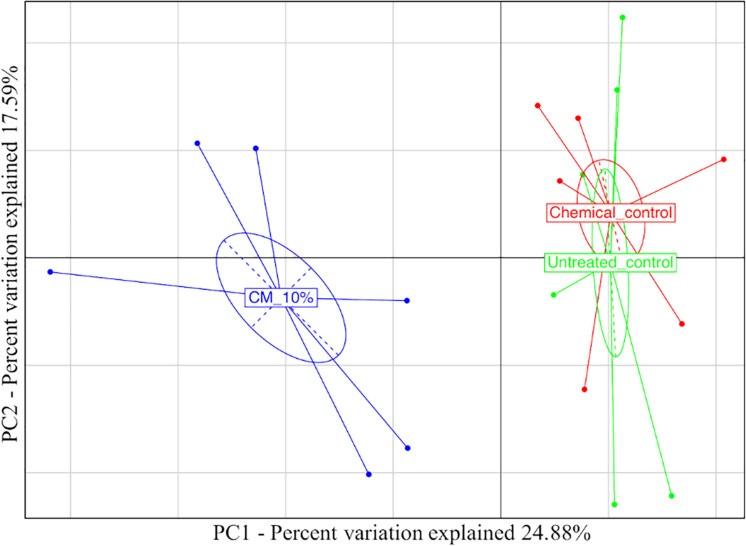
Principal component analysis based on the mycobiota composition referred to the rhizosphere samples at the end of the trials: untreated control (UC), chemical control (CC), and CM – 10% treatment. Three biological replicates were collected from three different pots per treatment and for each trial. The samples are color-coded according to the treatment.

**FIGURE 6:**
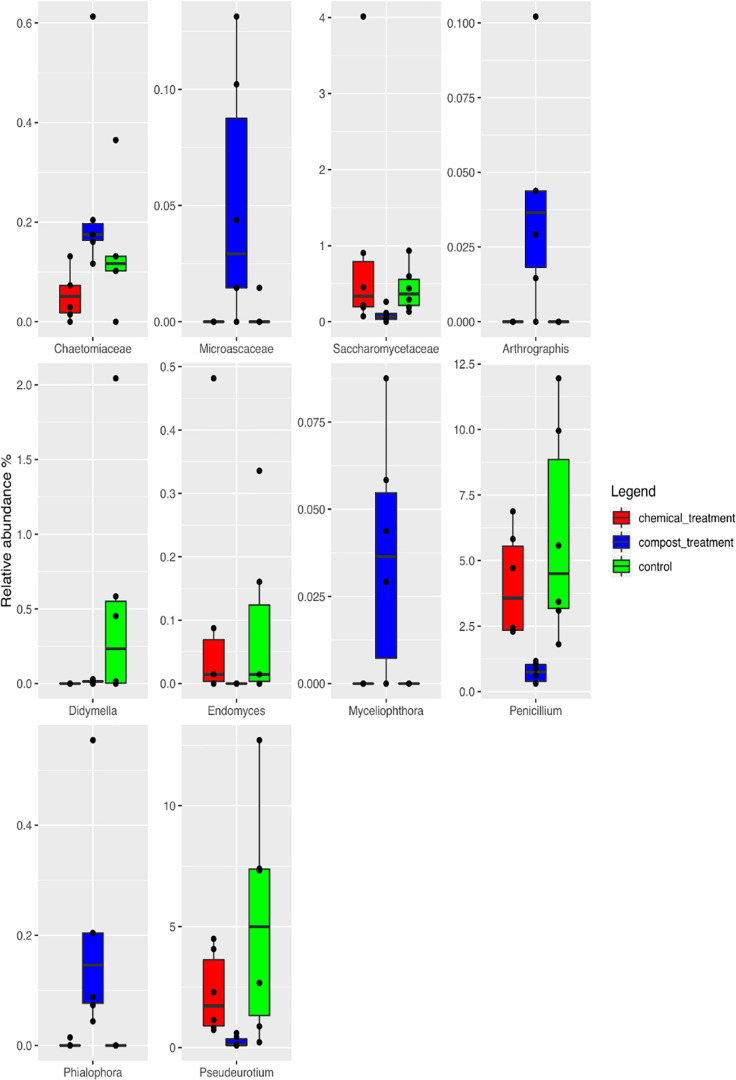
Boxplots showing the relative abundance of the differentially abundant OTUs based on the Wilcoxon matched pairs test (FDR ≤ 0.05) of the rhizosphere soil samples at the end of the trials: untreated control (UC), chemical control (CC), and CM – 10% treatment. Three biological replicates were collected from three different pots per treatment and for each trial. The boxes represent the interquartile range (IQR) between the first and third quartiles, and the line inside represents the median (2ND quartile). The whiskers denote the lowest and the highest values within 1.56 IQR from the first and third quartiles, respectively. The circles represent outliers beyond the whiskers.

Taking into account the microbiota composition at the highest taxonomic level (Figure 4 and Supplementary Table S2), it is possible to observe a core mycobiota, composed of *Fusarium*, which reaches about 3% of the relative abundance in the control samples (UC), 1% in the compost (CM – 10%) and 2% in the chemical treatment (CC); *Glomus*, which reaches 9, 5, and 15% in UC, CM – 10%, and CC, respectively; *Penicillium*, which reaches 6, 1, and 4% of the relative abundance in UC, CM – 10%, and CC, respectively; *Saccharomycetales*, which reaches 4, 1, and 3%; *Torrubiella*, which reaches 2, 2, and 1%; *Trichoderma*, which reaches 10, 12, and 7%; and *Zygoascus*, which reaches 1, 1, and 3%, respectively (Figure 4). A further separation of the samples, based on the treatment, was also observed, through the principal component analysis (PCA, Figure 5), and the result was confirmed by means of the ANOSIM statistical test (*P* = 0.003). Moreover, it was possible to observe a clear separation of the samples treated with compost, while the chemical treatment and control ones clustered together (Figure 5). By taking into account the significant difference in the OTUs among treatment (Figure 6, FDR < 0.05), it was possible to observe that the compost treatment (CM – 10%) was characterized by the presence of minor fraction OTUs. In other words, a higher presence of *Chaetomiaceae, Microascaceae, Arthrographis, Myceliophthora*, and *Phialophora* was observed (Figure 6), while *Penicillium* and *Pseudeurotium* were reduced in the compost treated samples, compared with UC and with CC. It should be observed that *Didymella* was reduced by both CM-10% and chemical treatments, compared to the untreated control (Figure 6).

### Co-occurrence Co-exclusion Analysis of the Mycobiota of the Rhizosphere Soils

**FIGURE 7:**
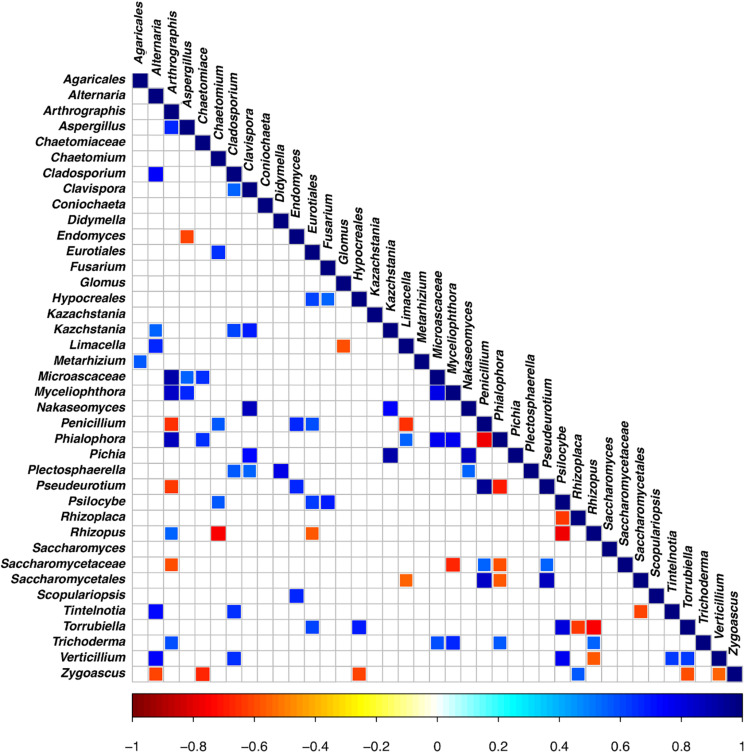
Significant co-occurrence and co-exclusion relationships between the OTUs of the rhizosphere soil samples at the end of the trials: untreated control (UC), chemical control (CC), and CM – 10% treatment. Three biological replicates were collected from three different pots per treatment and for each trial. The figure presents a Spearman’s rank correlation matrix (FDR < 0.01). The color of the scale bar denotes the nature of the correlation, with 1 indicating a perfect positive correlation (dark blue) and –1 indicating a perfect negative correlation (dark red).

The OTU co-occurrence/exclusion pattern of the rhizosphere soils is shown in Figure 7, where only significant correlations are reported (at a false discovery rate [FDR] of <0.01). As far as the main OTUs shared in the datasets are concerned, we observed that *Trichoderma* co-occurs with *Arthrographis, Myceliophtora, Phialophora*, and *Glomus*; *Verticillium* co-occurs with *Alternaria* and *Cladosporium*, while there is co-exclusion with *Rhizopus*; *Penicillium* shows co-exclusion with *Arthrographis*, *Limacella*, and *Endomyces*, while it co-occurs with *Chaetomium*; *Aspergillus* co-occurs with *Myceliophtora* and shows co-exclusion with *Endomyces*; *Alternaria* co-occurs with *Cladosporium*, *Limacella*, and *Verticillium*, while it co-excludes with *Zigoascus*; *Fusarium* co-occurs with *Hypocreales* and *Psillocybe.*

## Discussion

Four composts were analyzed to characterize their microbial community. Real-time PCR assays of the absolute abundance of genes have shown that all the four composts used in this study had a higher number of bacteria and fungi compared to literature (Epelde et al., 2018; Tortosa et al., 2020), confirming that all of them were potential sources of inoculum for agricultural applications. The mycobiota composition of the four compost analyzed showed a highest biological complexity.

In details we observed that CB showed several fungal genera that were less present in the other composts (such as *Scopulariopsis, Pseudeurotium*, and *Chaetomium*) highlighting the difference between green waste based and mixed waste based composts. A different mycobiota distribution was also observed among CV and CV2 probably due to the different procedures of composting. These aspects are in accordance with other studies that reported an implication of the composting procedures in the microbial modulation, especially when wastes were used (Anastasi et al., 2005; Neher et al., 2013). CV and CM were the same green composts, but CM was added with *Trichoderma* sp. TW2 strain; it is interesting to observe the difference, in terms of the relative abundance of the fungal genera, between these two composts which suggests that the composition of stable systems, such as a green commercial compost with a strong microbiome, can also be altered. The disease assessments showed that CM was the most effective; however, only CM – 10% was able to suppress the disease in the summer squash-PHC pathosystem and to reduce the disease incidence by 50% if compared to the untreated control. The fresh biomass and AUDPC were in accordance with the disease incidence in both experiments, and since no significative differences were found between the two trials, data were collapsed. CV, CV2, and CB were not effective in the protection of summer squash against PHC in any mixture concentration. This suggests that their microbial asset did not induce a modification of the rhizosphere microbial community able to establish the suppressive activity. As already reported by Bonanomi et al. (2007), composts suppressiveness is not a constant event. CV is the same compost as CM, but without the addition of the *Trichoderma* sp. TW2 strain, suggesting that the enrichment of composts with biological control agents may be a good strategy for this pathosystem. In addition, *Trichoderma* was effective against PHC, as already reported by many authors (Ahmed et al., 2000; Ezziyyani et al., 2007; Lorito et al., 2010; Bae et al., 2011). Furthermore, in a previous study carried out in field conditions (Gilardi et al., 2019), the application of *Trichoderma* TW2 alone against *Fusarium* wilt in lettuce was less effective compared to the use of CM compost, in combination with *Trichoderma* TW2.

Bonanomi et al. (2018) reviewed several works on biocontrol agents (BCAs) compost enrichment and pointed out that this could be the most promising way to achieve a long-term suppressiveness against soil-borne pathogens. In this context, the study of different composts, with the addition of different BCAs for several pathosystems, helps to clarify and identify the best one to use. On the other hand, BCAs can be selected by composts that showed a high suppressive action and can be used as inoculum to enhance the ability of other systems to suppress soil-borne pathogens (Pugliese et al., 2008). It is also important to underline that in this study, an excessive application of CM (20%) did not significantly suppress PHC disease incidence compared with the CM – 10%. This is in agreement with other papers (Bonanomi et al., 2007; Noble, 2011; Pugliese et al., 2011). The level of suppression is not always affected by compost dosage rate, and increased application rate only partially corresponds to increased disease suppression for composts (Bonanomi et al., 2007). Soil amended with ≥20%v/v of compost in 6 out of 79 experiments even showed a disease promotion effect (Noble, 2011). Furthermore, according to Pugliese et al. (2011), in non-sterilized composts, the addition of *Trichoderma* is not providing a dose-dependent effect against *Phytophthora*, as observed here. This dose-related effect of composts can be related to complex interactions among BCAs, compost’s initial microbiota and rhizosphere, which can explain why CM applied at the rate of 20% was not as effective as CM at 10%. Moreover, an occurrence of phytotoxicity due to the increasing rate of compost is also known (Bonanomi et al., 2007) and can explain the reduction in the efficacy of CM. Since only one compost mixture was suppressive against PHC, analyses of the rhizosphere of this treatment were carried out in order to establish whether the microbial communities were altered by the addiction of CM – 10%. Bacteria and fungi had almost similar gene copy number. This is in agreement with previous studies (Cucu et al., 2019), where the fungal and bacterial abundance in rhizosphere were found to be at the same level compared to bulk soil.

Real-time PCR of the total gene abundance showed no differences in the rhizosphere soils at the end of the trial for the CM – 10% treatment, chemical treatment, and untreated control for bacterial and fungal communities. Interestingly, PHC gene was found in the CM – 10% treatment and untreated control at similar levels, while it was not found in the chemical treatment, thus suggesting that the reduction in disease incidence in CM – 10% treatment was mediated by the complex interaction between *Trichoderma* and others microorganisms. The positive effect of CM – 10% treatment can also be due by the induction of resistance, which has already been reported to be stimulated by composts and *Trichoderma* spp. on many pathosystems (Vallad et al., 2003; Sang et al., 2010; Martínez-Medina et al., 2017; Savitha and Sriram, 2017). As for the mycobiota composition in the CM – 10% treatment, it was possible to observe that *Chaetomiaceae* and *Microascaceae* were higher than that in the untreated and chemical controls. *Chaetomiaceae* is a family composed of several genera, commonly found in air or soil environment (Rodríguez et al., 2002), that has already been related to beneficial actions, such as suppression of *Lisianthus Fusarium* wilt (Zhou X. et al., 2019), antibacterial activity (Chovanová and Zámocký, 2016), and putative entomopathogenic activity against Khapra Beetle (Mohammed et al., 2019). The high presence of *Microascaceae* in compost is not surprising, since this family has already been reported to increase during compost maturation (Galitskaya et al., 2017). Interestingly, the levels of *Penicillium* were lower in the CM – 10% compost treatment than in the chemical and untreated control, thus suggesting that this compost treatment could be able to reduce the presence of this genus, which is a well-known plant pathogen and mycotoxin producer (Olsen et al., 2019; Schmidt-Heydt et al., 2019; Vidal et al., 2019; Zinedine and El Akhdari, 2019). PCA analyses clearly showed a separation of CM – 10% treatment samples from chemical and untreated controls. The chemical control and untreated control clustered together in the PCA analyses, but the disease incidence was absent in the chemical control, thus suggesting that the fungicide used in this study was effective against *Phytophthora capsici* but did not alter the rhizosphere mycobiome, compared to the untreated control, while the CM – 10% treatment altered the equilibrium of the rhizosphere. This result is a further confirmation of the CM suppressive action conferred by the microbial community as a result of the interaction with *Trichoderma* sp. TW2. The co-occurrence and co-exclusion analyses highlighted that *Verticillium*, *Alternaria*, and *Cladosporium* occurred together, which is interesting because of the well-known pathogenic activity of these three genera (Rotem, 1994; Crous et al., 2007; Klosterman et al., 2009; Bensch et al., 2012). The fact that these three genera co-occurred in the rhizosphere samples suggests their potential cooperation in biotic stressed plants. In addition, we observed that *Trichoderma* co-occurred with *Glomus*, a genus considered the one that contains the highest number of arbuscular mycorrhizal species (Schwarzott et al., 2001). This suggests that a beneficial effect of *Trichoderma* is guaranteed in the rhizosphere environment, not only as a suppressive agent against *Phytophthora capsici* but also by improving the rhizosphere microbiome in terms of quality.

## Conclusion

This study involved four different composts analyzed for their microbial community composition and then used in a greenhouse pot trial in order to test their suppressive activity to prevent *Phytophthora capsici* infection against summer squash. The fungal community of the four composts were different, highlighting the central role of wastes choices and composting procedures in the selection of the mycobiota. Above 12 compost-peat mixtures, only CM – 10% was able to suppress PHC, due to its microbial composition that can play a major role in its suppressiveness (Reuveni et al., 2002; Tilston et al., 2002; Papasotiriou et al., 2013; De Corato et al., 2019). The mycobiota composition of the CM – 10% treated pots clustered separately if compared to CC and UC, confirming that beneficial microorganisms present in CM – 10% treatment can protect the plant root system by microbiota modulation (Antoniou et al., 2017; Mwaheb et al., 2017; Cucu et al., 2019; Topalović et al., 2020; Zhang et al., 2020). Further investigations should be necessary to obtain a deeper understanding of how this protection is conferred because the complex interaction between rhizosphere, microbiota and pathogens is less explored. Moreover, an activation of the systemic resistance of the host plants by CM – 10% treatment cannot be excluded. This study points out the importance of the exploration of microbial community of composts and their *in vivo* application to control soil borne diseases, in order to better understand how to predict the suppressive ability of a compost.

## Data Availability Statement

All sequencing data generated by this study can be found in the NCBI using accession number PRJNA580394.

## Author Contributions

AB, MP, and IF contributed to the conception and design of the study and writing of the original draft. AB and IF performed the statistical analysis. AB, MC, and IF contributed to the methodology and investigation. MP and MG contributed to reviewing. AB and MP contributed to editing. MP, MG, and AG supervised the study. MP contributed to the resources, project administration, and funding acquisition. All authors contributed to the article and approved the submitted version.

## Conflict of Interest

MP declares he has a financial interest as he is a shareholder in the AgriNewTech company that provided the products tested in this article. The remaining authors declare that the research was conducted in the absence of any commercial or financial relationships that could be construed as a potential conflict of interest.

## Supplementary Material

FIGURE S1 Boxplots to describe α-diversity measures of the rhizosphere soil samples at the end of the trials: Untreated control (UC), chemical control (CC), and CM – 10% treatment. Samples are color coded according to the treatment.
