# Supplementary figures and images for: A Compost Treatment Acts as a Suppressive Agent in Phytophthora capsici – Cucurbita pepo Pathosystem by Modifying the Rhizosphere Microbiota
Source: Front Plant Sci. 2020 Jun 24;11:885. doi: 10.3389/fpls.2020.00885 (PMC7327441; doi:10.3389/fpls.2020.00885)

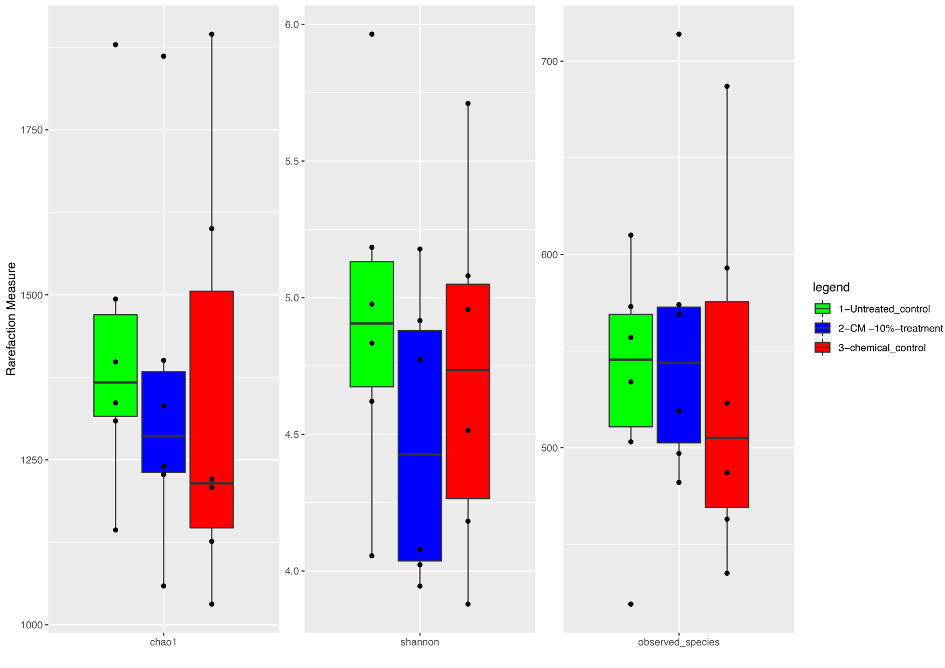

Supplement: FIGURE S1 — Boxplots to describe α-diversity measures of the rhizosphere soil samples at the end of the trials: Untreated control (UC), chemical control (CC), and CM – 10% treatment. Samples are color coded according to the treatment. [file Image_1.TIF]
