# Supplementary material for: A Compost Treatment Acts as a Suppressive Agent in Phytophthora capsici – Cucurbita pepo Pathosystem by Modifying the Rhizosphere Microbiota
Source: Front Plant Sci. 2020 Jun 24;11:885. doi: 10.3389/fpls.2020.00885 (PMC7327441; doi:10.3389/fpls.2020.00885)
Supplement: Supplementary file 2 [file Table_1.DOCX]

Table S1: Relative abundance of the fungal community in the four analyzed composts: CV green compost, CV2 green compost, CM green compost with the addition of *Trichoderma spp*. TW2 and CB mixed compost. The OTUs were selected by discarding the ones that were not present in the four composts under a threshold of 0.5 %.

| **Genera** | **CV** | **CV2** | **CM** | **CB** |
| --- | --- | --- | --- | --- |
| *Alternaria* | 0.03 | 0.15 | 0.01 | 0.61 |
| *Arthoderma* | 0.00 | 0.00 | 0.02 | 13.27 |
| *Aspergillus* | 0.03 | 0.59 | 0.19 | 4.16 |
| *Aureobasidium* | 8.46 | 0.07 | 0.30 | 0.24 |
| *Candida* | 0.01 | 0.00 | 0.02 | 0.00 |
| *Chaetomidium* | 4.28 | 0.14 | 0.32 | 0.16 |
| *Chaetomium* | 0.01 | 2.89 | 0.00 | 0.09 |
| *Cladosporium* | 0.57 | 0.55 | 0.07 | 5.27 |
| *Coniochaeta* | 2.47 | 0.00 | 0.00 | 3.33 |
| *Debaryomyces* | 0.02 | 0.02 | 0.04 | 0.15 |
| *Didymella* | 0.00 | 0.00 | 0.00 | 0.01 |
| *Fusarium* | 0.00 | 0.04 | 11.51 | 0.00 |
| *Galactomyces* | 0.62 | 0.03 | 0.04 | 0.10 |
| *Heterophoma* | 0.10 | 0.06 | 0.05 | 0.38 |
| *Kluyveromyces* | 0.42 | 0.03 | 0.02 | 0.12 |
| *Kurtzmaniella* | 0.06 | 0.00 | 0.00 | 0.02 |
| *Limacella* | 0.46 | 0.00 | 0.10 | 0.43 |
| *Lomentospora* | 0.01 | 0.00 | 0.01 | 1.10 |
| *Myceliophthora* | 0.43 | 0.01 | 0.32 | 4.78 |
| *Nigrospora* | 0.02 | 0.01 | 0.00 | 0.01 |
| *Penicillium* | 0.09 | 0.03 | 0.09 | 10.32 |
| *Phialophora* | 5.49 | 0.00 | 3.41 | 0.34 |
| *Pseudeurotium* | 0.00 | 3.97 | 0.00 | 2.46 |
| *Pseudozyma* | 0.01 | 0.01 | 0.00 | 0.00 |
| *Saccharomyces* | 0.00 | 0.00 | 0.02 | 0.00 |
| *Scopulariopsis* | 0.00 | 2.66 | 0.53 | 0.80 |
| *Tintelnotia* | 0.02 | 0.01 | 0.03 | 0.69 |
| *Trichoderma* | 0.00 | 0.00 | 6.39 | 0.06 |
| *Trichosporon* | 0.03 | 0.00 | 0.00 | 0.01 |
| *Wickerhamiella* | 0.00 | 0.02 | 0.00 | 0.03 |
| *Yarrowia* | 0.03 | 0.00 | 0.00 | 0.00 |
