# Supplementary material for: A Compost Treatment Acts as a Suppressive Agent in Phytophthora capsici – Cucurbita pepo Pathosystem by Modifying the Rhizosphere Microbiota
Source: Front Plant Sci. 2020 Jun 24;11:885. doi: 10.3389/fpls.2020.00885 (PMC7327441; doi:10.3389/fpls.2020.00885)
Supplement: Supplementary file 3 [file Table_2.DOCX]

Table S2: Relative abundance of the mycobiota in the rhizosphere samples at the end of the trials: Untreated control (UC), Chemical control (CC) and CM – 10% treatment. Three biological replicates were collected from three different pots per treatment and for each trial. Only OTUs which showed an incidence above 0.2% in at least 2 samples are shown. The data from replicates were averaged.

| **Genera** | **UC** | **CC** | **CM - 10%** |
| --- | --- | --- | --- |
| *Agaricales* | 1.52 | 1.64 | 0.87 |
| *Alternaria* | 0.07 | 0.08 | 0.25 |
| *Arthrographis* | 0.00 | 0.00 | 0.04 |
| *Aspergillus* | 0.06 | 0.04 | 0.87 |
| *Chaetomiaceae* | 0.14 | 0.05 | 0.24 |
| *Chaetomium* | 0.10 | 0.37 | 0.02 |
| *Cladosporium* | 1.06 | 0.10 | 0.28 |
| *Clavispora* | 0.09 | 0.04 | 0.02 |
| *Coniochaeta* | 0.01 | 0.02 | 0.02 |
| *Didymella* | 0.52 | 0.00 | 0.01 |
| *Endomyces* | 0.09 | 0.010 | 0.00 |
| *Eurotiales* | 0.02 | 0.01 | 0.03 |
| *Fusarium* | 3.37 | 1.60 | 1.45 |
| *Glomus* | 8.57 | 14.59 | 5.10 |
| *Hypocreales* | 0.31 | 0.26 | 0.60 |
| *Kazachstania* | 0.18 | 0.37 | 0.23 |
| *Kazchstania* | 0.68 | 1.16 | 1.77 |
| *Limacella* | 0.00 | 0.00 | 0.02 |
| *Metarhizium* | 0.23 | 0.10 | 0.05 |
| *Microascaceae* | 0.11 | 0.11 | 0.10 |
| *Myceliophthora* | 0.00 | 0.00 | 0.04 |
| *Nakaseomyces* | 0.11 | 0.11 | 0.06 |
| *Penicillium* | 5.97 | 4.08 | 0.73 |
| *Phialophora* | 0.00 | 0.00 | 0.20 |
| *Pichia* | 0.45 | 0.64 | 0.74 |
| *Plectosphaerella* | 2.75 | 0.00 | 0.05 |
| *Pseudeurotium* | 5.20 | 2.26 | 0.24 |
| *Psilocybe* | 1.41 | 0.338 | 0.56 |
| *Rhizoplaca* | 0.43 | 0.14 | 0.15 |
| *Rhizopus* | 0.07 | 0.12 | 0.51 |
| *Saccharomyces* | 0.03 | 0.10 | 0.09 |
| *Saccharomycetaceae* | 0.43 | 0.98 | 0.09 |
| *Saccharomycetales* | 4.14 | 3.48 | 1.45 |
| *Scopulariopsis* | 0.58 | 0.16 | 0.04 |
| *Tintelnotia* | 0.01 | 0.00 | 0.07 |
| *Torrubiella* | 1.67 | 0.67 | 1.53 |
| *Trichoderma* | 9.84 | 7.30 | 11.66 |
| *Verticillium* | 0.10 | 0.04 | 0.10 |
| *Zygoascus* | 1.25 | 2.84 | 0.61 |
